# Expression of activator protein-1 in papillary thyroid carcinoma and its clinical significance

**DOI:** 10.1186/s12957-019-1568-x

**Published:** 2019-01-31

**Authors:** Cheng Xiao, Yonglian Huang, Qiyuan Gao, Zijian Feng, Qi Li, Zhen Liu

**Affiliations:** Department of Pancreatic & Endocrine Surgery, Shengjing Hospital of China Medical University, No. 36 Sanhao Street, Heping District, Shenyang, Liaoning Province China

**Keywords:** Activator protein-1, Papillary thyroid carcinoma, Immunohistochemistry

## Abstract

**Background:**

The abnormal expression of activator protein-1(AP-1) has recently been investigated in a variety of tumors. While the relationship between AP-1 and thyroid cancer is poorly studied, our study was to evaluate the protein expression and clinical value of AP-1 in papillary thyroid carcinoma (PTC).

**Methods:**

The expression of AP-1 was examined by immunohistochemistry on paraffin-embedded tissues obtained from PTC and correspondent paracancerous tissues of 82 patients.

**Results:**

Compared with paracancerous tissues, AP-1 expression was significantly elevated in PTC tissues and the positive rate was 79.3% (65/82). Our study found a linear trend relationship between the expression of AP-1 and tumor size. However, the differences in AP-1 expression among gender, age, lymph node metastasis, number of lesions, location of the lesion, and extrathyroid invasion are not statistically significant.

**Conclusions:**

The expression of AP-1 plays an important role in the proliferation process of PTC.

## Background

Thyroid cancer is one of the most common malignancies of the endocrine system. The incidence of thyroid cancer has continued to rise in the past several decades worldwide [1]. In 2013, there were 143,900 new cases of thyroid cancer reported and 6500 deaths in China, the national incidence of thyroid cancer was 10.58 per 100,000 (5.12 per 100,000 for men and 16.32 per 100,000 for women), and the ratio between males and females was 1:3.2 [2]. Thyroid papillary carcinoma is the most common type and contributes to more than 85% of thyroid cancer [3]. Previous studies have established that excessive activation of the MAPK pathway can drive carcinogenesis in BRAF, RAS, and RET gene mutation induced by PTC, and activator protein-1 (AP-1) is an important target of the MAPK pathway [4, 5].

AP-1 is a leucine zipper protein dimer that is composed of Jun (c-Jun, JunB, and JunD), Fos (c-Fos, FosB, Fra-1, and Fra-2), ATF (ATF-2, LRF1/ATF-3, B-ATF), JDP (JDP-1, JDP-2), and Maf (c-Maf, MafA, MafB, MafG/F/K, Nrl) [6]. Jun protein is able to form homodimer by itself; it also can form heterodimer with Fos and ATF protein family members. However, Fos protein cannot form homodimer with Jun protein. Jun-Fos dimer is the most common form of AP-1 protein in human cells. AP-1 is a downstream transcription factor of the MAPK signaling pathway that binds to specific DNA sequences on other genes, participating in a wide range of cellular processes, including cell growth, differentiation, and apoptosis [7]. Growth factors, neurotransmitters, cytokine, stress, ultraviolet, and other physiological factors can activate the transcription factor AP-1 via the MAPK signaling pathway and increase the transcriptional activity of Jun and Fos. The post-transcriptional phosphorylation and dephosphorylation of Jun and Fos can regulate the process of cell proliferation, invasion, metastasis, survival, and apoptosis [8]. When AP-1 is inappropriately expressed, it is closely related to pathological processes such as tumor cell transformation, angiogenesis, metastasis, immunity, and inflammatory diseases [9, 10]. It has been shown that nasopharyngeal cancer tissues have increased expression of AP-1 compared with normal tissues, and it was related to the progression of tumor cells [11]. Blocking the transcriptional activation of AP-1 suppresses the process of breast cancer cell invasion [12]. Some studies have also reported that the expression of AP-1 protein is upregulated in various tumor tissues such as pancreatic cancer [13] and colon cancer [14]. However, the mechanisms of AP-1 and papillary thyroid carcinoma are not well studied. The purpose of our study is to evaluate the expression and clinical significance of AP-1 in papillary thyroid carcinoma.

## Methods

### Tissue samples and patients

Cancer tissues and correspondent paracancerous tissues were obtained from 82 patients with PTC who underwent thyroid surgery. The histologic sections were reviewed by two expert pathologists to verify the histologic diagnosis. All samples were obtained from Shengjing Hospital of China Medical University from July 21, 2011, to July 21, 2017. Inclusion criteria were cases with complete clinical data and pathological tissue specimen. All patients in this study had signed an informed consent that their clinical and pathological data be used for research purposes. The study protocol was approved by the Clinical Research Ethics Committee of the Shengjing Hospital of China Medical University.

### Immunohistochemistry

Three-micrometer-thick, formalin-fixed sections were prepared and deparaffinized, and gradient alcohol hydration, endogenous peroxidase activity was blocked with 3% hydrogen peroxide for 15 min. Antigen retrieval was performed by boiling the slides for 8 min in citric acid buffer (pH 6.0), then cooled to room temperature. Slides were incubated with the rabbit anti-human polyclonal AP-1 antibody (dilution, 1:640; proteintech, China), at 4° overnight. Slides were rinsed with PBS and incubated with secondary antibody (goat anti-rabbit, Zhongshanjinqiao, Beijing, China) at room temperature for 30 min, stained with DAB(3,3-diaminobenzidine) (Fuzhou Maixin, Fujian, China) solution for 1–2 min, counterstained with hematoxylin, dehydrated, coverslipped, and analyzed by fluorescence microscopy. AP-1-positive staining is localized in the cytoplasm and nuclear. Known positive sections were used as the positive control, and PBS buffer was used instead of primary antibody as the negative control group.

Five high-power fields were randomly collected from each slice under an optical microscope and scored by two clinical pathologists. Immunohistochemical localization of AP-1 protein was cytoplasm and nucleus. The extent of staining was estimated on a scale of 0 to 4 (0, none; 1, < 10%; 2, 10~ 50%; 3, 51~ 80%; 4, > 80%). The intensity of staining was scored on a scale from 0 to 3 (0, no staining; 1, weak; 2, moderate; 3, strong). The immunoreactive score of AP-1 was determined by the product of extent and intensity. The score ≥ 2 was considered positive expression.

### Statistical analysis

Statistical analysis software SPSS 24.0 (SPSS, Inc., Chicago, IL, USA) was used for data analysis. Chi-square test was used to analyze the differences of AP-1 protein expression between PTC and paracancerous tissues. The relationship between AP-1 protein expression and clinical data characteristics in PTC was evaluated by chi-square test and Fisher’s exact probability. The relationship between tumor size and AP-1 protein expression was analyzed by assessing the linear-by-linear association results in trend chi-square test of SPSS. *P* < 0.05 was considered as statistically significant.

## Results

### The expression of AP-1 protein in thyroid tissue

The study group included 82 patients (17 males, 65 females), with an age range of 18~77 years (mean ± SD = 41.0 ± 12.5 years). There were 61 patients with lymph node metastases, and 29 patients with extrathyroid invasion. The remaining patients’ characteristics are shown in Table 1.

**Table 1:** Relationship between AP-1 expression and clinicopathological features in papillary thyroid carcinoma

Clinical features	*n*	AP-1 expression [cases (%)]		*P* value
		Positive	Negative	
Gender				
Male	17	11 (64.7)	6 (35.3)	0.184
Female	65	54 (83.1)	11 (16.9)	
Age				
≥ 55	10	10 (100)	0 (0)	0.19
< 55	72	55 (76.38)	17 (23.62)	
Tumor size				
≤ 2 cm	56	40 (71.43)	16 (28.57)	0.012
> 2 cm, ≤ 4 cm	25	24 (96)	1 (4)	
> 4 cm	1	1 (100)	0 (0)	
Lymph node metastasis				
Yes	61	50 (82)	11 (18)	0.473
No	21	15 (71.4)	6 (18.6)	
Multifocality				
No	42	33 (78.6)	9 (21.4)	0.873
Yes	40	32 (80)	8 (20)	
Location of lesions				
Single side	59	48 (81.4)	11 (18.6)	0.086
Bilateral	23	17 (73.9)	6 (26.1)	
Extrathyroid invasion				
Yes	29	25 (83.2)	4 (16.8)	0.657
No	53	40 (75.5)	13 (24.5)	

Clinical characteristics of patients, and comparisons between expression of AP-1 and tumor size

**Fig. 1:**
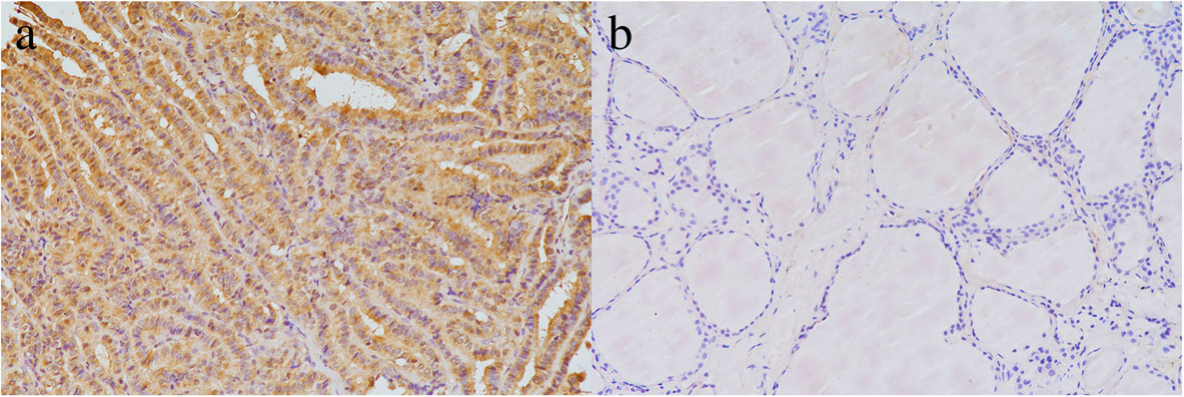
Immunohistochemical staining for AP-1 in PTC and paracancerous tissues. **a** PTC tissue with AP-1 staining (magnification, × 200). **b** Paracancerous tissue without AP-1 staining (magnification, × 200)

Under light microscopy, AP-1 protein was mainly expressed in the nucleus and/or cytoplasm of PTC and paracancerous normal tissues. No expression was found in the intercellular substance. The positive expression of AP-1 protein in PTC was 79.3% (65/82), which was significantly higher than paracancerous tissues (26.8%, 22/82). There was a significantly increased expression of AP-1 protein in PTC (*P* < 0.05) (showed as Fig. 1a and Fig. 1b).

### Associations of AP-1 protein expression with clinical features in papillary thyroid carcinoma

AP-1 expression was significantly positively correlated with tumor size by using the trend test (*P* = 0.012). But it was negatively associated with the patient’s age, gender, number of lesions, location of lesions, lymph node metastasis, and extrathyroid invasion (*P* > 0.05).

**Fig. 2:**
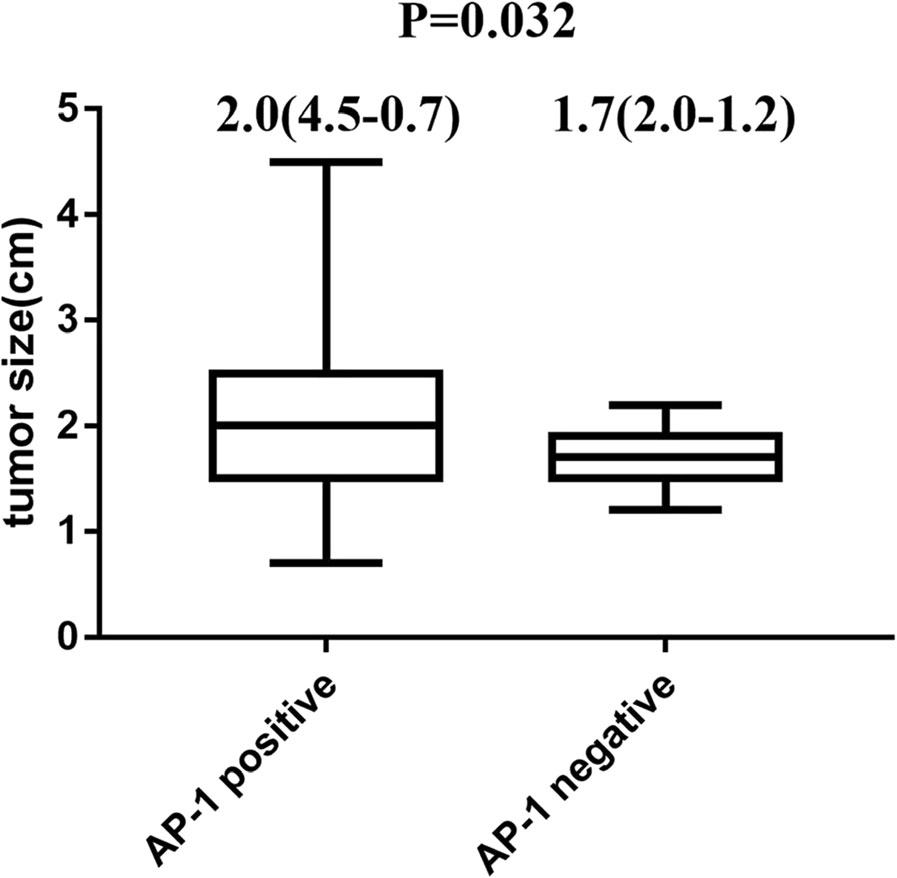
Relationship between the expression of AP-1 and tumor size

As shown in Fig. 2, AP1-positive patients exhibited significantly larger tumor size than AP1-negative patients [2.0 (4.5–0.7) cm, *n* = 65 vs. 1.7 (2.0–1.2) cm, *n* = 17, *P* = 0.032].

## Discussion

AP-1 is a leucine zipper protein that is assembled through the dimerization of a characteristic bZIP domain (basic region leucine zipper) in the Fos and Jun subunits. Numerous studies have reported that the activation of transcription factor Jun and c-fos can induce the expression of cyclinD1. CyclinD1 is a member of the cyclin protein family that is involved in increasing DNA synthesis and accelerating cell cycle progression. The synthesis of cyclinD1 drives the cell cycle progression from G0/G1 phase to S phase in tumor proliferation [15]. Ming et al. showed that IL-7 could induce cyclinD1 gene expression via an AP-1(c-Fos/c-Jun)-dependent pathway and promote lung cancer cell proliferation [16]. AP-1 activation also drives VEGF (vascular endothelial growth factor) expression and regulates the process of proliferation in blood endothelial cells and various tumor cells [17].

Previous study also reported that the expression of c-Jun, JunD, and Fra-1 protein significantly increased in human thyroid cancer tissue and played a critical role in the process of thyroid cancer cell proliferation [18]. However, Chen et al. suggested that the expression of AP-1 was negative to the tumor size [19]. We found that the tumor size of AP-1-positive group was larger than that of the negative group (*P* < 0.05). By using the trend test in SPSS, we found the expression of AP-1 protein increased with tumor size in PTC (*P* = 0.012). Some studies have reported that tumor size can predict persistence, recurrence, and death [20, 21]. PTC persistence was defined as evident structural and/or biochemical residual disease until 1 year after initial surgery. Disease detected after 1 year was considered as PTC recurrence. In a retrospective analysis with a 10-year follow-up PTC cohort study [22], the author reported that tumor size was a predictor of PTC persistence. Following the enlargement of tumor size, the risk of persistence increased. A long-term study of 1355 patients with thyroid cancers demonstrated that tumors smaller than 1.5 cm had lower 30-year recurrence and lower cancer mortality rates than those larger ones [23]. These data suggested that AP-1 may serve as a key factor in PTC cell proliferation. And it may also be used as a predictor for prognosis of PTC.

AP-1 is not only closely related to the process of tumor proliferation, but also influences the local invasion and distant metastasis of tumors by regulating VEGF and matrix metalloproteinase 9 (MMP-9). By using AP-1 transcription inhibitors, the proliferation and invasion of VEGF-dependent vascular endothelial cells in tumor cells could be blocked [24]. In addition, the transcription factor AP-1 can also regulate the expression of MMP-9 by binding to the MMP-9 promoter [25]. Upregulation of MMP-9 expression can degrade various components of the extracellular matrix and destroy the histological barrier, causing the tumor progression and invasion [26, 27]. AP-1 and NF-κB are overexpressed in esophageal cancer cells. The increasing transcriptional activity of MMP9 contributes to the invasion and metastasis of esophageal cancer [28]. In a study of 66 patients with PTC and 40 patients with benign thyroid tumors, VEGF and MMP-9-positive expression were more frequently seen in PTC and were closely correlated to tumor size and clinical stage [29]. Because of the relationship between MMP-9, VEGF, and AP-1, we guessed that expression of AP-1 contributed to the lymph node metastasis of PTC. However, our study did not find any significant difference between AP-1 expression and lymph node metastasis.

In many tumors, the oncogene Ras continuously activates and phosphorylates JNK through the MAPK signaling pathway. Activation of the transcription factor AP-1 upregulates the expression of MMP-9 and VEGF and promotes invasion and metastasis of tumor cells. On the other hand, JNK is also reported as a tumor suppressor in different types of cancer. JNK promotes apoptosis by phosphorylating c-Jun. Then, c-Jun activates the Fas apoptotic protein pathway and protease Caspase-3 to initiate apoptosis [30]. Meggiato et al. [31] found that c-jun and Caspase-3 were highly expressed on human pancreatic cancer. There was also a significant correlation between caspase3 and c-Jun. Shaulian and Karin [32] showed that JunB upregulated the expression of tumor suppressor genes and decreased cyclinD1 expression. Mitsiades et al. [33] demonstrated that bortezomib phosphorylated and activated c-Jun through the JNK signaling pathway, which initiated the Fas apoptosis pathway and improved prognosis by promoting apoptosis of medullary thyroid carcinoma and undifferentiated thyroid cancer cells. These results indicated that c-jun/AP-1 had a bidirectional effect on cell proliferation or apoptosis. In our study, there was no evident correlation between AP-1 expression and lymph node metastasis. It was consistent with Chen’s study. But the reason was still not clear.

In conclusion, our study demonstrated that the level of AP-1 protein was significantly upregulated in PTC compared with paracancerous thyroid tissue by immunohistochemistry. The expression of AP-1 is positively correlated with tumor size. Previous study identified that tumor size was a predictor of prognosis of PTC. AP-1 may serve as an outcome predictor due to the trend relationship between AP-1 and tumor size. These results showed that the immunohistochemical evaluation of the level of AP-1 in PTC might be useful as a molecular marker for the diagnosis and prognosis of PTC. And AP-1 activation may serve as a potential factor involved in the proliferation and transformation of PTC. However, the detailed mechanism of AP-1 in lymph node metastasis, extrathyroid invasion, and apoptosis regulation in PTC has not been clarified due to its complex composition. Further studies need to be focused on this promising protein.
